# Conceptual model of low-cost improvised bubble continuous positive airway pressure device for adults and its potential use in the COVID-19 pandemic

**DOI:** 10.1371/journal.pntd.0010221

**Published:** 2022-03-03

**Authors:** Himal Kharel, Zeni Kharel, Samikchhya Keshary Bhandari

**Affiliations:** 1 Sukraraj Tropical and Infectious Disease Hospital, Kathmandu, Nepal; 2 Rochester General Hospital, Rochester, New York, United States of America; 3 Maharajgunj Medical Campus, Kathmandu, Nepal; University of Cape Town Faculty of Health Sciences, SOUTH AFRICA

## Abstract

Low-cost improvised continuous positive airway pressure (CPAP) device is safe and efficacious in neonatal respiratory distress. There is a great necessity for similar device in adults, and this has been especially made apparent by the recent Coronavirus Disease 2019 (COVID-19) pandemic, which is unmasking the deficiencies of healthcare system in several low-resource countries. We propose a simplified and inexpensive model of improvised CPAP in adults using locally available resources including aquarium air pumps and a novel pressure release mechanism. Although the safety and efficacy of improvised CPAP in adults are not established, the conceptual model we propose has the potential to serve as a lifesaving technology in many low-resource settings during this ongoing pandemic and thus calls for expedited research.

## Author summary

An aquarium air pump, few plastic tubes, and a tight-fitting mask can be used to form an improvised continuous positive airway pressure (CPAP) device that could be lifesaving for many in low-resource countries. Although the device seems promising, further research is needed to establish its safety and efficacy.

## Introduction

Bubble continuous positive airway pressure (BCPAP) has been proven to be a safe and efficacious mode of noninvasive ventilation for neonatal respiratory distress. Commercially available adult continuous positive airway pressure (CPAP) machines are expensive to purchase and maintain, making them inaccessible to a large population of low-resource countries. The need for such devices has especially been realized during the current Coronavirus Disease 2019 (COVID-19) pandemic, which is ravaging the fragile health system of several low-income countries. Hence, we propose a conceptual model of improvised CPAP that could have widespread applications in low-resource settings.

## Conceptual design

**Fig 1:**
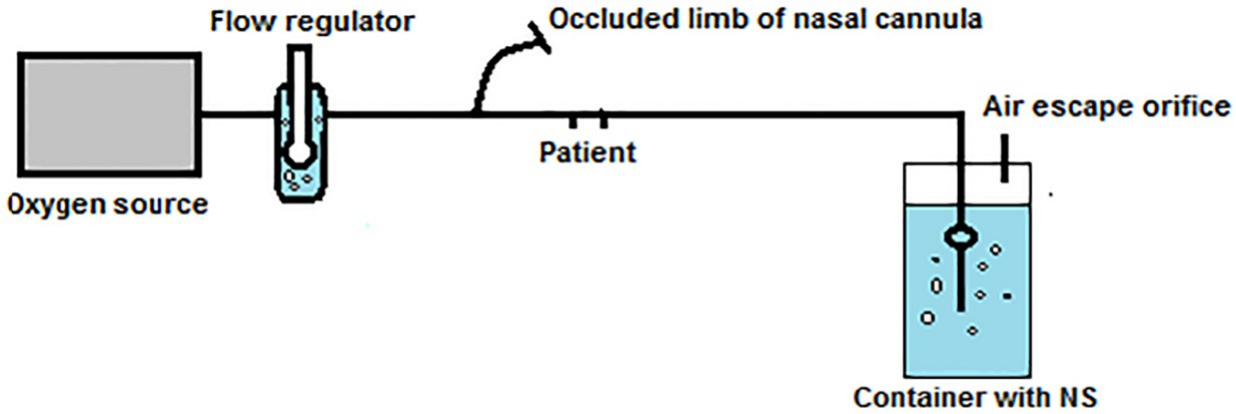
A simplified neonatal BCPAP circuit. BCPAP, bubble continuous positive airway pressure; NS, Normal Saline.

A basic form of functional BCPAP for neonates consists of a nasal cannula, normal saline bottle, measuring tape, and an oxygen source. One of the limbs of nasal cannula is cut and occluded, while the other end is dipped into normal saline. The depth of submersion determines the amount of CPAP. A 14-gauge intravenous cannula is inserted into the normal saline bottle to function as air escape orifice. The oxygen flow rate is adjusted to achieve gentle bubbling [1]. This is illustrated in Fig 1.

In order to generate positive pressure during inhalation, the flow rate in the device must exceed the rate at which tidal volume is inhaled. As neonates have only a fraction of the adult tidal volume, oxygen flow rate of 5 to 10 L/min is usually sufficient. One of the main problems encountered while creating a similar device for adults is that the flow rate of oxygen required exceeds the flow rate that can be maximally provided by the wall outlets of low-resource settings. This means that the flow rate of oxygen must be increased if BCPAP is to be used for adults. This can be done using air pumps or by using Venturi devices that entrain room air. However, Venturi devices are unable to generate a constant pressure. Rather, the only time they are able to generate positive pressure is during the exhalation [2]. Hence, the most feasible way to generate constant positive airway pressure is by using air pumps.

**Fig 2:**
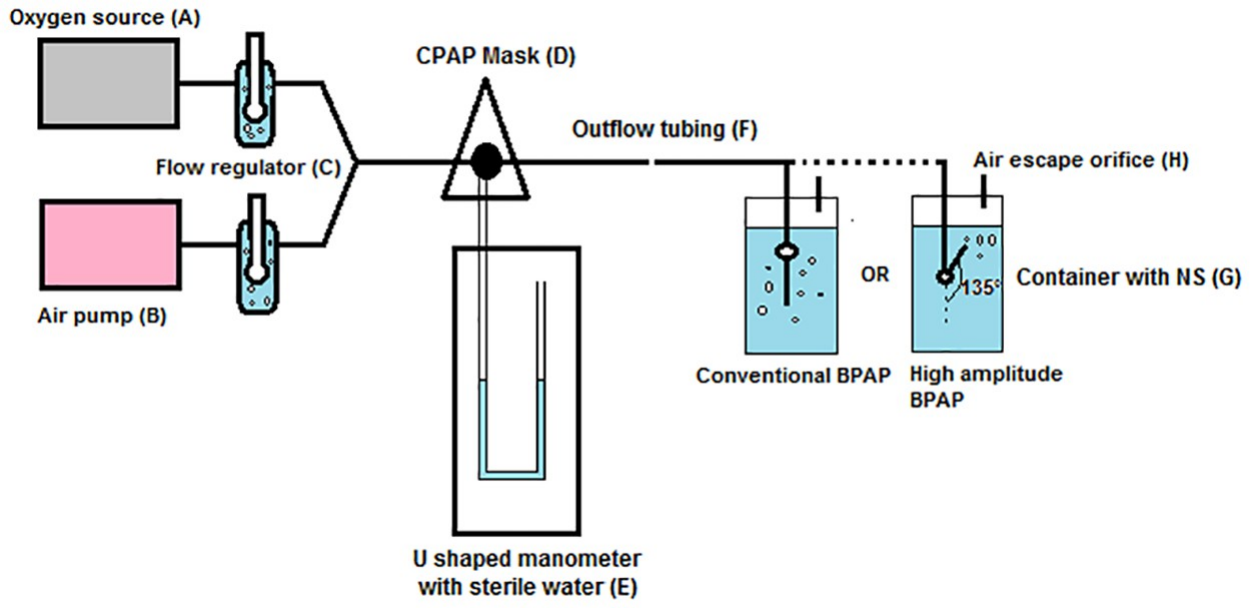
A simplified model of modified improvised BCPAP for adults. BCPAP, bubble continuous positive airway pressure; CPAP, continuous positive airway pressure; NS, Normal Saline.

**Fig 3:**
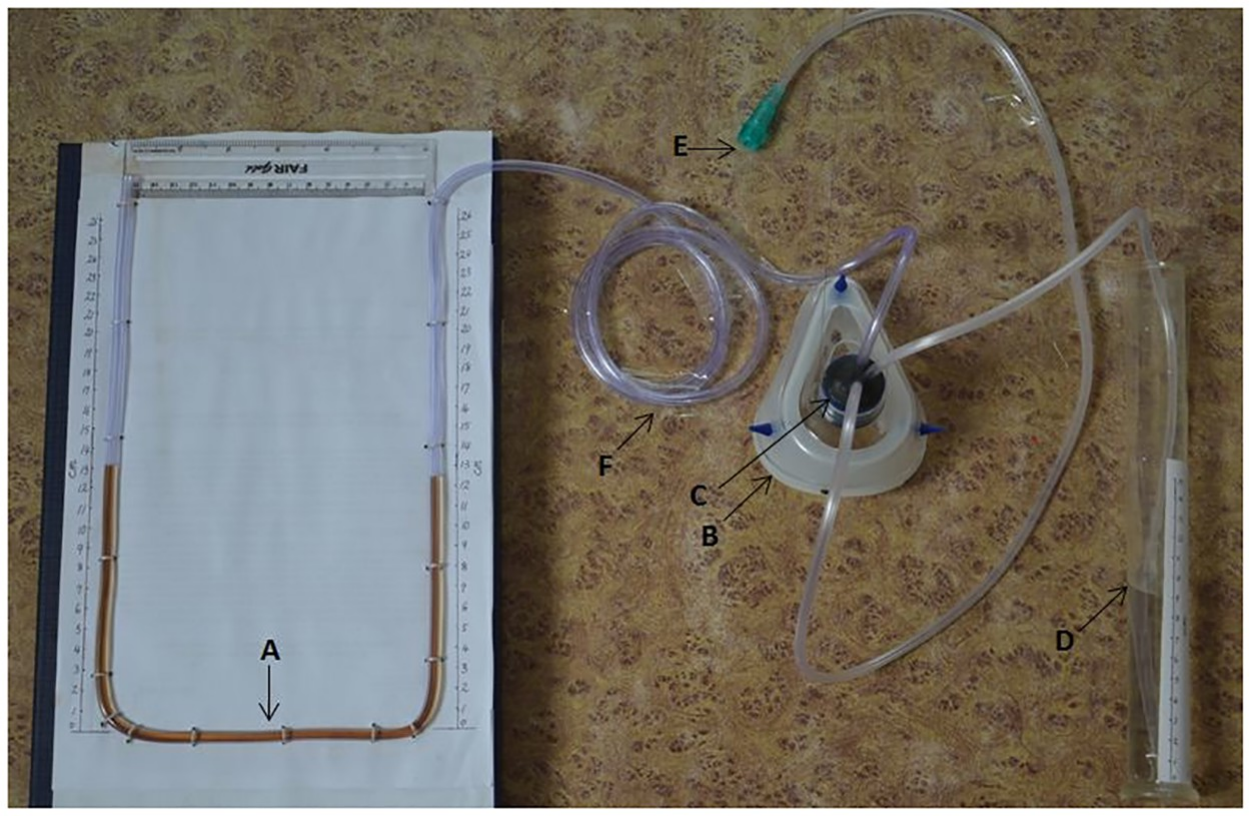
A mock model demonstrating U-shaped manometer filled with (A) dyed normal saline, (B) tight-fitting face mask, (C) fast-curing epoxy putty, (D) container containing NS, (E) port through which blended air can be passed, (F) and plastic tubing. NS, Normal Saline.

Our model has been conceived by combining and modifying many aspects of models proposed by Milliner and colleagues, Daga and colleagues, and Brown and colleagues [3–5]. Our model is illustrated in Fig 2. A mock-up device showing the mask, its connections, and the novel pressure releasing mechanism is shown in Fig 3.

Our model consists of the following:

(A) Oxygen source: Wall oxygen or oxygen concentrator can be used as the oxygen source.
(B) Aquarium air pump: Our model utilizes aquarium air pump to provide compressed air as it is cheap and has been used in the past [4,5].
(C) Flow regulator with humidifier: Two flow regulators with humidifiers on the oxygen source and aquarium air pump each allow for control of the fractional inspired oxygen and flow rate of blended air. The flow rate must be greater than the rate at which patient inhales the tidal volume. For example, if patient inhales 400 mL tidal volume in 2 seconds, the flow rate must be greater than 200 mL/s or 12 L/min. Oxygen concentration of the blended air and the blended air flow rate can be estimated with the following formula:
$$Oxygen\;concentration\;of\;blended\;air(vol\%)=\;\frac{\left\{0.21\times air\;flow\;rate+1.0\times oxygen\;flow\;rate\right\}\times \left\{\left(P_{atm}-P_{H20}\right)/760\right\}}{air\;flow\;rate+oxygen\;flow\;rate},$$
  where P _atm_ is the atmospheric pressure, and P _H2O_ is the vapor pressure of water at 37 °C.
  Unlike oxygen concentration of the blended air, the pressure provided by the device doesn’t need correction for the atmospheric barometric pressure, vapor pressure of water, and ambient temperature. This is because our device is an open system that has free communication with the environment, and the pressure experienced by the patient is a relative pressure rather than an absolute pressure.
(D) CPAP face mask: Nonleaky CPAP face mask is essential for proper functioning of this system. Mask of bag and mask ventilation system can also be used. The tube with blended air, tube connecting to U-shaped manometer, and outlet tube are inserted into the opening in the face mask and sealed using fast-curing epoxy compound (Bond Set 2-in-1, Resinova Chemie, Kanpur, India). Adhesive tape can also be used for this purpose.
(E) Pressure release mechanism: The presence of this mechanism is essential for the safe functioning of improvised CPAP device to prevent dangerously high airway pressures if the main tubing is accidentally blocked. A piece of paper fastened with a rubber band to the CPAP circuit has been proposed theoretically by Milliner and colleagues to act as a pressure release mechanism [3]. However, the bursting strength of paper will be altered by the moisture of humidified air. We propose an alternative in which a locally constructed U-shaped manometer can act both as pressure sensor and pressure releaser. A tube can be made into a U shape and fixed onto a plyboard. The afferent limb attaches to the mask, while the efferent limb is open to the external atmosphere. For example, if we want to release pressure above 30 cm of water, we must construct an U-shaped tube with the efferent limb of at least 30 cm and fill it with water such that 15 cm of fluid is present on each of the afferent as well as efferent limbs. Once the pressure exceeds 30 cm of water, the blended air will start to bubble and escape from the U-shaped manometer, preventing excess buildup of pressure.
(F) Outflow tubing: The outflow tubing must be at least 8 mm in internal diameter. Above this threshold, pressure generated by the system is independent of tube length and oxygen flow rate as demonstrated by Mestriner and colleagues [6].
(G) Sterile water/normal saline container: The outflow tubing is submerged into sterile water or normal saline container with the amount of submersion determining the amount of CPAP.
(H) Air escape orifice (H): Air escape orifice of at least 8 mm is necessary for creating CPAP, which is independent of oxygen flow rates as demonstrated by Mestriner and colleagues [6].
(I) Altering the angle of bubble outflow: The outflow tubing of conventional CPAP is immersed vertically into container “G.” High-amplitude BCPAP can be generated by altering the angle of bubble outflow relative to vertical line to 135 degrees. This part is optional.

## Discussion

Noninvasive CPAP may be beneficial in COVID-19–associated acute hypoxemic respiratory failure. A retrospective single-center study found that CPAP prevented intubation and mechanical ventilation in 36 out of 53 patients with moderate to severe acute hypoxemic respiratory failure possessing gas exchange and computerized tomography (CT) scan findings that were severe enough to warrant intubation and mechanical ventilation [7]. Similarly, another study found that CPAP is beneficial in avoiding excessive burden on intensive care unit resources by significantly increasing intubation-free survival [8]. Unfortunately, noninvasive CPAP machines are costly, and compressed air supply is not readily available in low-resource settings. Improvised CPAP, however, can be constructed cheaply with minimum resources.

In contrast to traditional ventilator-generated continuous positive airway pressure (VCPAP), the physical process of bubbling in BCPAP creates high-frequency and low-amplitude oscillations in the airway pressure, which simulates high-frequency ventilation in neonates [1]. Not only that, these high-frequency oscillations have been shown to increase carbon dioxide clearance in preterm infants [9]. The advantage of BCPAP over VCPAP in neonates has been well demonstrated in terms of length of hospital stay, number of complications, and cost [1]. However, similar studies in adults are lacking, and there is no evidence regarding generation of similar oscillations in adults.

Diblasi and colleagues found that alteration of the angle of exit of bubbles in relation to vertical line in BCPAP had huge influence in altering the amplitude and frequency of pressure oscillations seen in the airways of juvenile rabbits [10]. The amplitude of pressure oscillations increased, while the frequency of oscillations decreased significantly when the vertical exit angle of bubbles was changed to 135 degrees. This was hypothesized to be due to stochastic resonance. It means counterintuitive enhancement of periodic respiratory fluctuations when superimposed over the “noise” created by the physical process of bubbling. This modification of BCPAP is called high-amplitude BCPAP. It demonstrated decreased breathing effort among neonates compared to standard BCPAP; however, adult studies are lacking in this regard [11].

In addition, this modification significantly decreased interleukin 6 (IL-6) and total protein in bronchoalveolar lavage fluid in ventilator-induced lung injury model in adult rats [12]. IL-6 has been implicated as one of the chief mediators responsible for acute respiratory distress syndrome (ARDS) in COVID-19. Tocilizumab, an anti-IL-6 receptor monoclonal antibody, has been used in the treatment of severe COVID-19 [13]. We hypothesize that high-amplitude BCPAP may alter the natural history of COVID-19 ARDS by reducing IL-6, one of its chief mediators. There is one pilot study conducted on 10 healthy volunteers and one published case report in which similar device has been used successfully in a pulmonary tuberculosis (TB) patient with type 1 respiratory failure [3,14]. Similar to COVID-19, IL-6 plays a significant role in the pathogenesis of TB. It is the major cytokine elevated in vitro in *Mycobacterium tuberculosis*–infected mouse peritoneal cells, and its concentration decreases after treatment of TB [15]. So, anecdotal efficacy of BCPAP in acute hypoxemic respiratory failure due to TB may be transferrable to ARDS in COVID-19, although more studies are essential to further explore this hypothesis.

The lack of measures to prevent aerosol generation is one of the drawbacks of our model. Use of helmet as delivery device also doesn’t seem to be viable as such devices are difficult to be found in low-resource settings. One of the ways to prevent viral dissemination when using masks is the use of electrostatic filters. However, the placement of electrostatic filters is not a feasible alternative for the purpose of our model. Davis and colleagues found that the mean time to failure point of electrostatic filters for neonatal BCPAP ranged from 257 to 525 minutes [16]. We can assume that the failure time for adult CPAP will be significantly less considering the high airway pressure required for adults and subsequent generation of more aerosols and moisture. In addition, the mean pressure generated was also significantly greater in systems that used such filters [16]. This means requirement of frequent change of filters and further difficulty in maintaining the safe pressure in the system.

The healthcare systems in several low-resource countries are on the verge of collapse due to explosive increase in COVID-19. Even though the evidence regarding safety and efficacy of improvised BCPAP in COVID-19 patients is currently unknown, this paper highlights a potential area of research that could be extremely beneficial during these trying times.
